# Severe Hyperammonemia in Late-Onset Ornithine Transcarbamylase Deficiency Triggered by Steroid Administration

**DOI:** 10.1155/2015/453752

**Published:** 2015-04-09

**Authors:** Jordi Gascon-Bayarri, Jaume Campdelacreu, Jordi Estela, Ramon Reñé

**Affiliations:** ^1^Unitat de Diagnòstic i Tractament de les Demències, Neurology Service, Hospital Universitari de Bellvitge, Feixa Llarga s/n, 08907 L'Hospitalet de Llobregat, Spain; ^2^Neurology Service, Hospital Universitari Parc Taulí, Parc del Taulí 1, 08208 Sabadell, Spain

## Abstract

Ornithine transcarbamylase deficiency (OTCD) is a rare X-linked disorder of urea synthesis leading to hyperammonemia. Several late-onset cases have been reported. Undiagnosed and untreated patients are at the risk of death or suffering from irreversible sequelae. We describe a 56-year-old patient who presented with acute encephalopathy after steroid treatment. Hyperammonemia due to OTCD was diagnosed and a mutation was found. This allowed us to diagnose two other family members with unexplained encephalopathy who are now asymptomatic on a low-protein diet. OTCD should be considered in any patient with hyperammonemic encephalopathy and immediate treatment should be given to avoid a fatal outcome. We emphasize the need to examine other family members if the diagnosis is confirmed, in order to prevent further life-threatening episodes of encephalopathy or neonatal coma of newborn.

## 1. Introduction

Ornithine transcarbamylase deficiency (OTCD) is a rare X-linked disorder of urea synthesis leading to hyperammonemia [1] that usually presents in newborn. An increasing number of late-onset cases have been reported in adults, most of them females [2, 3] with symptom onset coinciding with a precipitating factor [4]. However, in some cases the trigger cannot be identified [5]. Without treatment patients may die or suffer from irreversible cognitive sequelae [1]. It is therefore crucial to identify these late-onset patients and to test the rest of the family if a mutation is found.

## 2. Case Presentation

A previously healthy 56-year-old Spanish man was admitted due to acute disorientation and agitation. He had suffered a common cold 3 weeks before. Four days prior to admission he had received a steroid injection to treat his knee arthritis. He had a decreased level of consciousness (Glasgow Coma Scale 9) but a normal physical examination. He required sedation and orothracheal intubation. Blood tests revealed mild leukocytosis (14,46 × 10^9^/L) and high ammonium levels (320 *μ*g/dL), with normal pH, liver, and renal function, negative serologies and antibodies (HSV, HIV, autoantibodies, and antineuronal antibodies), and normal CSF (biochemistry, cell count, HSV PCR, culture). Brain CT and MR were normal. EEG showed a severe encephalopathy with acute bifrontal activity. A mycoplasma serology was positive and parainfectious encephalitis was suspected because of the previous cold. A urea cycle disorder triggered by drugs was also considered and amino acids were measured in blood and urine, showing no relevant abnormalities. Urine orotic acid was not measured. The clinical picture and the ammonium levels resolved after hemodialysis.

Two months later he underwent a laser cordotomy and required steroid treatment because of glottic edema. Four days later he developed progressive encephalopathy and required orothracheal intubation again. Blood tests revealed hyperammonemia and leukocytosis; the rest of the blood and CSF tests were again normal. OTCD was suspected and DNA analysis revealed a c.622G>A (p.A208T) mutation. The patient improved after hemodialysis and has remained asymptomatic.

**Figure 1:**
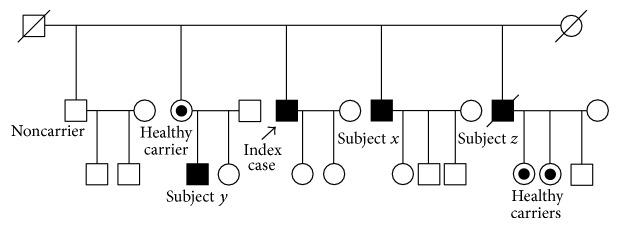
Family tree. Subjects *x* and *y* are affected carriers. Subject *z* has two carrier daughters and is suspected to have been an affected carrier.

Other family members were studied (Figure 1). Two of them had been suffering from episodes of encephalopathy and could be finally diagnosed. Subject *x* (brother) is a 55-year-old male, who had complained of excessive somnolence after meals for 3 years previously, which resolved after starting a low-protein diet; he was recently admitted because of hyperammonemic encephalopathy triggered by infection and steroid treatment, which resolved after hemodialysis. He had a normal basal amino acid profile and urine orotic acid. The diagnosis was genetically confirmed. Subject *y* (nephew) had suffered several episodes of hyperammonemic encephalopathy from the age of 13; the first episode had been attributed to metoclopramide, the following one to juvenile epilepsy, and the last ones to azathioprine treatment for Crohn's disease. Since OTCD was confirmed two years ago, he has followed a low-protein diet. He has normal ammonia and amino acid levels and has not suffered any other episodes. Subject *z* (brother) had died with encephalopathy and brain edema of an unknown cause. He was a donor and his liver was rejected for transplantation because of its pathological macroscopic appearance. His two daughters are mutation carriers, but not his wife, so the patient must have been affected.

## 3. Discussion

Patients with late-onset OTCD are frequently misdiagnosed as encephalitis, poisoning, psychotic illness, or epilepsy. A personal or family history of similar episodes and precipitating factors should be investigated [4]. Hyperammonemia can be triggered by high protein intake, increased protein catabolism due to infection, trauma, physical exercise or steroids, hepatic toxicity from chemotherapy and other medications, pancreatitis, or postpartum [5–8]. There are at least five reports of OTCD adults who developed acute hyperammonemic coma following steroid administration [3, 6, 9–11]. Glucocorticoids are known to have a general catabolic effect by primarily enhancing protein turnover [12].

Ammonia should be measured in patients of any age presenting an unexplained encephalopathy and a urea cycle defect should be suspected if the patient has a normal liver function. Elevated glutamine and alanine plasma levels, decreased citrulline, and presence of orotic acid in the urine are usually found in OTCD [13], but these can be within normal range in the compensated state, as was the case of subject *x*. These tests should be performed in the acute phase, and if this is not possible, samples should be frozen for further testing. Regardless of the results, if the suspicion of OTCD is high, a genetic analysis should be performed.

Mutation analysis is the method of choice for the definitive diagnosis. In the majority of patients the mutation appears* de novo* (mother not carrier). In almost 20% of OTCD patients the mutation is not identified [14, 15]; OTC enzyme activity assay in liver or intestinal mucosa can help in such cases. The measure of urinary orotidine excretion in women after the administration of a single dose of allopurinol (allopurinol challenge test) is also a simple and reliable method of assigning carrier status to women suspected of carrying a mutation [16].

A review in 2006 identified 341 mutations of the OTC gene [15] and new ones are being discovered (http://www.biobase-international.com/product/hgmd). The mutation found in our family has been already reported in other families [17, 18], with a variable age of onset and presentation, from asymptomatic to fatal coma.

Hemodialysis is the treatment of choice for rapid reduction of ammonia levels in adults and should be started quickly, even before confirming the diagnosis, in order to prevent intracranial hypertension and cerebral edema. Other measures include intravenous hydration with glucose and lipids, ammonia scavengers,* L*-arginine, and cessation of protein intake [6, 19]. Long-term management is based on a low-protein diet. Liver transplantation may be considered in selected patients [14].

The possibility of an OTCD should be considered in any patient with hyperammonemic encephalopathy and treated rapidly to avoid fatal coma or long-term cognitive sequelae. We emphasize the importance of measuring ammonia and considering the diagnosis of urea cycle disorders in any patient with encephalopathy. The other family members need to be examined if the diagnosis is confirmed because it is a treatable condition and therefore further life-threatening episodes of encephalopathy or neonatal coma of the newborn can be appropriately prevented and rapidly treated.
